# Effectiveness of a mobile phone application in managing voice health for teachers: A randomized controlled trial

**DOI:** 10.1177/20552076251351805

**Published:** 2025-07-02

**Authors:** Jonas Hauck, Christian Gegner, Marina Giglberger, Sven Hilbert

**Affiliations:** 1 Department for Educational Science, 9147 University of Regensburg, Regensburg, Germany; 2 Department of Applied Speech Science, 9147 University of Regensburg, Regensburg, Germany; 3 Department of Psychology, 9147 University of Regensburg, Regensburg, Germany

**Keywords:** Prevention, voice health, voice disorders, mHealth, eHealth, teachers, app, randomized control trial, smartphone

## Abstract

**Objective:**

Teachers are at constant risk of straining their voices due to professional demands, making them more susceptible to voice disorders and therefore increased absenteeism. A widely accessible prevention method would be beneficial, leading to the development of the ReSt mobile application. The aim of this study was to evaluate its effectiveness.

**Methods:**

A randomized controlled trial was conducted with 399 teachers, allocating 205 participants to the training group and 194 to the control group. Over a 46-day period, participants in the training group engaged with the ReSt app. Outcomes were assessed using self-reports via the Voice Handicap Index (VHI-12) and objective evaluations employing the Dysphonia Severity Index (DSI). Additionally, app use was fully tracked using log data to gain insight into user engagement.

**Results:**

On average, the training resulted in substantial improvements in the DSI within the training group compared to the control group ( γ=2.22,SE=0.32,p<.001 ). No training effect was observed concerning the VHI-12 ( γ=−0.39,SE=0.46,p=.388 ). The average number of days spent opening exercises in the app was 10.57 days (SD = 9.07), and time spent on exercise-related app screens averaged 2.38 hours (SD = 2.79).

**Conclusions:**

The app appears to have promising effects on physiological measured voice capability, but more training time may be needed to minimize the psychosocial impact of voice problems. Our results mark an important advancement in voice disorder prevention approaches, especially given previously inconclusive evidence for traditional voice training methods.

## Background

The human voice is an important tool for communicating with others. In our modern, communication-oriented society, members of various professions rely to varying degrees on their voices to do their jobs. A distinction is drawn between non-occupational voice users (e.g., administrators, librarians, and chemists) and occupational voice users (e.g., actors, singers, teachers, and call center agents).^1^ For the latter group, voice disorders are particularly serious because they can limit professional work or even make it impossible. Voice disorders are referred to as dysphonia, which is “characterized by altered vocal quality, pitch, loudness, or vocal effort that impairs communication and/or quality of life.”^2^ Occupational voice users have a 2–4 fold higher risk of developing a voice disorder during the course of their career compared to non-occupational voice users.^3^ Teachers are a particularly large group who, unlike actors and singers, are often not adequately prepared for their vocal profession.

### Assessment of voice

Voice is a multifactorial product that can be perceived as an acoustic phenomenon. Air exhaled from the lungs causes the vocal folds in the larynx to vibrate. This source signal is converted into sound by shaping the vocal tract (the space between the vocal folds and the lips).^4^ Voice diagnostics attempt to examine all areas and mechanisms involved in voice production and to identify any perceived impairment or effects caused by a voice disorder on the affected person. Several approaches exist^5,6^: self-perception (e.g., the Voice Handicap Index [VHI-12]),^7^ auditory perception of the voice,^8,9^ acoustic analysis of the voice signal,^10^ aerodynamic measurements,^11^ and laryngeal endoscopic imaging.^12^ Whereas the first two methods are based on perception and are subjective, the last three methods require instrumental equipment and can be considered objective. However, since imaging procedures must also be interpreted by an examiner, these are sometimes regarded as subjective procedures.^13^

### Prevalence of dysphonia in teachers

A meta-analysis indicates that the prevalence of voice disorders among teachers varies widely across countries.^14^ An international comparison shows heterogeneous rates: Malaysia reports 10.4% ( N=6039 ),^15^ whereas Brazil exhibits 69.1% ( N=439 ).^16^ German investigations identified pathology in 25% of teachers ( N=536 ).^17^ Historical data aligns with these ranges, with Australia documenting 19% prevalence ( N=1168 )^18^ and US studies indicating 57.7% among teachers ( N=1243 ).^19^ Methodological variations, including temporal parameters and diagnostic instruments,^3,14,20^ contribute to these disparities. Assessment approaches range from self-report questionnaires^18,19^ to standardized instruments like the VHI^15,16^ and clinical evaluations.^17^ Whereas this methodological heterogeneity impedes cross-national comparisons, empirical evidence consistently demonstrates elevated voice pathology risk in the teaching profession.

### Risk factors

From a biopsychosocial perspective, voice health is influenced by the interaction of physical, mental, and social factors.^21^ In general, a voice disorder can arise when the demands placed on the vocal apparatus exceed its functional capacity. This may result from external (environmental) factors that impose strain on the voice, as well as individual factors such as inadequate vocal technique or concurrent medical conditions.^22^ Environmental risk factors for teachers include number of pupils,^23,24^ noise level in the classroom,^24^ student discipline problems,^25^ classroom temperature,^24^ prolonged voice use,^25^ using a loud voice,^26^ subject,^27^ grade level,^28^ years in occupation,^19^ high workload,^23^ and work-related stress.^23,24^ Individual factors include general health, concomitant diseases, muscular tension,^23,25,29^ previous voice problems,^23^ family history of voice disorders,^24^ gender,^19,24,25,30^ age,^19^ and lifestyle habits such as alcohol consumption.^29^ Although previous studies have come to different conclusions regarding risk factors, a meta-analysis of 16 studies between 2004 and 2018 identified the following main risk factors: “gender, upper airway problems, caffeine consumption, speaking loudly, number of classes per week, and resignation experience due to voice problems.”^31^

### Impact of voice disorders

In teachers, voice disorders have a negative impact on overall well-being^32^ and are often associated with mental health problems (e.g., major depressive episode and general anxiety disorder).^30^ Because those affected experience their professional performance as limited,^33^ the result may be a desire for career reorientation.^33^ Moreover, high absenteeism,^34^ presenteeism,^35^ and the cost of treating teachers with voice disorders are a burden on the healthcare system.^36^ In the USA, the annual cost has been estimated at USD 2.5 billion.^37^ In addition, voice disorders in teachers appear to have an impact on students. The primary symptom of a voice disorder is a modification in vocal quality, such as hoarseness. Studies show that hoarse voices can not only lead to negative attitudes among students,^38^ but may have a negative impact on students’ cognitive performance and, thus, on the learning process.^39–41^

### Prevention programs

Given the elevated risk of voice disorders among teachers throughout their careers and the extensive consequences associated with such conditions, there is a growing call for the implementation of preventive programs. These include general voice training programs during teachers’ education or career,^23,26,34,42,43^ early detection,^25,44,45^ and consideration of acoustic conditions.^42,46^ Although individual studies confirm the effectiveness of direct training (direct vocal tract training),^47^ indirect training (vocal hygiene guidelines that influence voice production),^48^ and a combination of trainings,^47,49^ a meta-analysis of 12 studies from 2004 to 2021 found no significant effects.^50^ Potential reasons contributing to this absence of effect may include variations in study methodologies (with some studies focusing on self-perception whereas others examined acoustic or aerodynamic conditions of the voice), the different sample sizes ( N=9 to N=286 ), and shortcomings in the study design (e.g., no control group). Therefore, a study is needed with a sufficiently large sample, a control-group design, and a comprehensive assessment of the voice to establish conclusive evidence on the efficacy of preventive training interventions for teachers.

## The ReSt app

In addition to these methodological challenges, there are also practical barriers to implementing traditional face-to-face preventive interventions, including logistical and resource constraints. Mobile health applications offer a promising solution for delivering scalable voice training interventions. In Germany, there is a manageable selection of eHealth applications for voice and speech, which are not scientifically evaluated and have different areas of focus. These include apps for singing (Amsel), specialized therapy and musicality training apps (SpeechCare), and tools for lecturers and speakers (Besser Sprechen), as well as offerings aimed more at teachers, such as the web-based voice training solution (Körper-Stimme-Haltung, University of Halle-Wittenberg) and the blended learning offering (LEHGU, Freiberg Institute for Musicians’ Medicine). Nevertheless, there is still a gap in the market for mobile applications designed specifically for teachers. For this reason, the ReSt app is designed to help teachers maintain a healthy voice despite their teaching activities. This mobile phone application offers 18 pages of background information on how the voice and speech work, as well as 55 awareness exercises and training sessions covering all areas of voice production: posture and tone, breathing, phonation, and articulation. There are exercises to promote good muscle tone in the body, relaxation techniques such as progressive muscle relaxation, dream journeys, deep breathing exercises, phonation tube exercises, exercises to relax and train the articulation muscles, and exercises to build resonance. The app also features 15 practical teaching tips and resources for dealing with voice-related problems. The knowledge and exercise content is multimodal, alternating between images, text, video, and audio. To increase compliance, the app offers the option to set fixed training times, receive push notifications for mindfulness nudges, save favorites and use them offline, as well as gamification aspects (e.g., different levels and progress indicators for each area).

## Methods

The aim of this study was to evaluate the effectiveness of the ReSt mobile application in improving teachers’ voice health. We formulated the following hypotheses:

1. After using the ReSt mobile application, the training group will show a significant improvement in self-reported voice health, as indicated by lower scores on the VHI-12, compared to the control group.
2. Physiological voice parameters, as measured by the Dysphonia Severity Index (DSI), will improve significantly in the training group compared to the control group, with higher DSI scores reflecting better voice health.

Additionally, the mobile Application Usability Questionnaire (MAUQ) was analyzed to evaluate the usability of the application as a secondary analysis without associated hypothesis.

The study was approved by the ethics committee at the University of Regensburg, Germany (*Ethikkommission der Universität Regensburg*^1^; date: 11/23/2022; number of approval: 2-3123-101), and the protocol, including materials, procedures, and statistical analyses, was pre-registered in the Open Science Framework (OSF) prior to completion of data collection (osf.io/537gz).

### Study design

A randomized controlled study design was used, with a 1:1 allocation ratio of training to wait-listed control (8-week delay). The study was carried out at a single research site in Regensburg, Germany.

### Study sample

The study sample consisted exclusively of practicing teachers, with no pre-service teachers. The participants represented a wide range of educational settings, including primary schools, the three different types of German secondary schools (*Mittelschule*, *Realschule*, and *Gymnasium*), special needs schools (*Förderschulen*), and vocation-oriented schools in Bavaria. Special needs schools are specialized in serving students with attention deficits, learning challenges, and possible developmental disorders, while adhering to the standard academic curriculum. Vocation-oriented schools function as traditional secondary schools but incorporate specialized coursework focused on occupational training and career-specific subjects. For analytical purposes within this study, both the special needs schools and vocation-oriented schools were classified under the secondary school category, as their student populations fell within comparable age ranges.

Recruitment was conducted through events, newsletters, and collaboration with school administrators. Individuals who met the following criteria were excluded: current voice or speech therapy, current psychotherapy, not using an iPhone or Android phone. No financial compensation was provided for participation. However, all teachers received feedback on their completed questionnaires and, where applicable, on their voice assessments.

Effect sizes were calculated prior to the study using the pwr package^51^ in R.^52^ Our target sample size was N=300 participants, as this is realistic for such a study and would provide sufficient power to detect small mean effects ( δ> 0.42) between the post-test scores of the target variables with a power of 1−β>.95 .^53^ Due to high logistic effort and limited personnel resources, sample size was smaller for the physiological voice parameters (voice assessment; n=100 ), meaning that effects must be larger ( δ> 0.73) to be detectable with a power of 1−β>.95 . However, due to the direct effect of the mobile application on these parameters, larger effects are expected.

### Randomization and blinding

Randomization was conducted by the first author, who had the least direct contact with participants. We employed a stratified cluster randomization approach using computer-generated sequences via the cvrall() command from the R package cvcrand.^54^ This method prospectively assigned participants to either the training group (TG) or the control group (CG), with all participants from the same school allocated to the same group (cluster) to prevent information exchange about the app. The allocation sequence was designed to ensure balanced distribution across both school types (primary or secondary) and participation in the voice assessment. Blinding was not implemented in this study because all researchers were required to provide different study materials to the teachers in the CG and TG, such as instructions for installing the ReSt app, and, therefore, needed to be aware of each individual’s group allocation. Blinding of participants was also not possible in this study. However, participants were not explicitly told of their allocation to either TG or CG. Rather, they were informed that access to the ReSt app would be granted upon completion of either the pre- or post-test assessment. This modified blinding approach represents a pragmatic compromise given the inherent limitations of the study.

### Procedures

During the recruitment phase, prospective participants were provided with detailed information regarding the objectives and procedures of the study. Teachers from the local area were offered a standardized in-person voice assessment. Teachers who lived and worked further away from the study site could not be offered this opportunity due to the logistical constraints.

Participants who provided informed consent and did not meet any of the exclusion criteria were randomized. At the pre-test assessment, all participants were required to complete an online questionnaire via a self-hosted LimeSurvey service (community edition version 6.2.6, LimeSurvey GmbH, Hamburg, Germany), which collected data on demographic and occupational characteristics, personal physical and mental health status (e.g., depression, anxiety, or chronic physical diseases), and voice use habits. For those who opted in at enrollment, the voice assessment was conducted within five days of completing the questionnaire. This voice screening was carried out by five people according to a standardized protocol, which is available in the OSF project (osf.io/b3nd7).

Participants in the TG were granted unrestricted access to the ReSt app for a 46-day period. To ensure controlled usage, the app was not made publicly available through platforms such as the App Store or Google Play; instead, participants received individual download links. At the end of the trial period, access to the app automatically expired. After completing the training, TG participants repeated the online questionnaire, which excluded demographic and occupational questions but included items assessing satisfaction with the ReSt app. If a pre-test voice assessment was administered, a post-test voice assessment was scheduled within 5 days of completing the post-test questionnaire. Participants received feedback on their responses and assessment results only after the study was fully completed. The CG followed an identical protocol, with the exception that their use of the ReSt app occurred after the post-test rather than after the pre-test assessment.

The study employed staggered start dates across participating schools between 18 September 2023, and 2 February 2024, addressing both logistical constraints and strengthening the research design. This approach mitigated potential influence factors (such as work-intensive periods for teachers) while accommodating each school’s scheduling needs. Despite the varied start dates, the duration of app usage remained consistent for all participants in the TG. The complete schedule for all cohorts is available in the Supplemental Material in the OSF project.

### Measures

The two pre-specified primary outcome measures were self-reported and objectively measured voice health. Self-reported voice health was assessed at baseline and after 46 days using the widely used and validated German version of the VHI-12.^7^ This index is designed to measure intrapsychic, social, and communicative consequences resulting from voice problems.^7^ The instrument comprises 12 items assessing voice-related symptoms and their impact on daily functioning. Each item is rated on a 5-point Likert scale (0 = never, 4 = always). The total score ranges from 0 to 48, with higher scores indicating greater voice-related impairment and diminished quality of life. The VHI-12 score enables classification of voice health severity according to established reference values.^55^ Scores are interpreted as follows: 0–6 indicates normal voice function, 7–13 reflects mild impairment, 14–22 suggests moderate impairment, and scores above 22 represent severe voice impairment.

Objectively measured voice capability was assessed during both pre- and post-test voice assessment using the validated DSI,^56^ a key component of instrument-based phoniatric assessment. The DSI is calculated based on four components with different weighting: the highest possible frequency in the singing voice, the lowest intensity in the singing voice, maximum phonation time (maximum duration of the vowel “a”), and jitter (pitch fluctuation).^56^ These four parameters, out of a total of 13, were identified as the most predictive of the severity of auditorily perceived dysphonia.^56^ A normal, healthy voice can be characterized by a score of 3.30 or higher,^57^ although other values can be found in the literature.^58^ The DSI, with its measures closely linked to vocal capacity, is considered to reflect limitations in vocal function. It can serve as a global measure of vocal performance or capability,^59^ with higher values indicating better vocal function. The DSI was measured with the standardized registration program LingWAVES (version 3.2, WEVOSYS Medical Technology GmbH, Bamberg, Germany, 2017). The program was run on two comparable computers, both equipped with identical external sound cards and pre-calibrated external sound level meters to ensure measurement accuracy. The distance between the sound level meter and the subject’s mouth was ∼ 30 cm, and the subject was instructed to maintain the same position throughout the procedure.

App usability and satisfaction were assessed using a translated version of the mHealth App Usability Questionnaire (MAUQ, see the supplemental material),^60^ administered with the other post-test questionnaires to the TG only. The MAUQ is an 18-item scale with a 7-point Likert scale (0 = strongly disagree, 6 = strongly agree). In addition to an overall usability score, it evaluates three specific domains of usability: ease of use (MAUQ_E, five items), interface and satisfaction (MAUQ_I, seven items), and usefulness (MAUQ_U, six items).

In the context of application usage, participant activity was fully logged using the open-source web analytics application Matomo (version 4.15.1, InnoCraft Limited, New Zealand). Data were collected as time-stamped event logs, with each row representing a registered event (e.g., clicking on an activity, scoring an activity, and pausing a video). These events were tagged with contextual information about the relevant activity or chapter. Based on this data, we calculated the total time spent on exercise-related screens in the app and the number of days exercises were visited in the app. To ensure measurement accuracy, we implemented a threshold of 18.5 minutes for exercise duration—a value specifically chosen to align with the longest designed activity within the application. This threshold helped filter out longer sessions that likely represented instances where participants left their devices with screens active rather than actual engagement. The app usage variables were directly measured rather than self-reported, ensuring a high degree of objectivity.

### Statistical analyses

All analyses were conducted using the statistical software R (version 4.4.2 “Pile of Leaves”).^52^ The app data were imported using the jsonlite package,^61^ and data processing was handled with the tidyverse package suite.^62^ For statistical modeling, we employed the lme4 package,^63^ whereas p -values were computed with the lmerTest package.^64^ Model fit assessment was performed using the MuMIn package.^65^ The results were formatted and organized using the packages kableExtra^66^ and broom.mixed.^67^ Missing data occurred when participants dropped out before completing baseline or exit assessments, despite required fields. Training access required initial questionnaire completion, reducing but not eliminating baseline missing data. The linear mixed models employed are advantageous in handling such missing data patterns, as they can accommodate missing values without necessitating the exclusion of entire cases, unlike traditional variance analyses approaches.^68^

Two linear mixed models were estimated to evaluate the training’s effectiveness, building upon the methodology of Hilbert and colleagues.^68^ Both models were applied separately to DSI and VHI-12 measures, resulting in four analyses in total. Although our preregistration proposed a single model, we opted for separate models per dependent variable to enhance interpretability of the training effects. The first model incorporated three fixed effects: *sampling point*, *group*, and their interaction, along with *age*, *gender*, and *workload* as covariates. Random intercepts were included to control for varying baseline measurements across participants. The second model focused exclusively on the TG and examined two key metrics of app engagement: *total app usage in hours* and *unique days with app usage*, while maintaining the same covariates and random intercepts. Both *app hours* and *app days* were analyzed as level-2 variables to properly account for the hierarchical nature of our data. To assess model fit, both marginal $R^{2}$ (representing the variance explained by fixed effects) and conditional $R^{2}$ (accounting for both fixed and random effects) were calculated.^69^

All analytical code and datasets have been made publicly accessible through the OSF project. Statistical significance was established at the more conservative p=.01 due to the larger number of linear mixed models employed, implementing a more stringent threshold than pre-registered to mitigate alpha error inflation.

## Results

### Participants

A total of N=399 teachers completed the consent form, met the inclusion criteria, were randomized, and started at least the first questionnaire ( $n_{CG}=194$ , $n_{TG}=205$ ). The voice assessment had high participation, with n=196 teachers opting to participate ( $n_{CG}=101$ , $n_{TG}=93$ ). A total of 189 participants in the CG and 203 participants in the TG completed the pre-test VHI-12. For the post-test VHI-12, 179 participants in the CG and 176 participants in the TG completed the assessment. In the TG, 174 teachers completed the post-test MAUQ. At pre-test, DSI completion numbers were 98 (97%) participants in the CG and 91 (98%) in the TG. Software crashes and user errors resulted in three CG participants having only a post-test DSI score. Post-test completion numbers were 80 (79%) participants for the CG and 70 (75%) for the TG, with attrition primarily due to illness-related absences.

Table 1 presents the demographic characteristics of the study participants. In line with other educational research, the sample was predominantly female, reflecting the gender distribution of teachers in the German school system. Participants ranged in age from 23 to 65 years. The teachers had a mean teaching experience of 14.6 years ( SD=9.0 , range: 1–37 years) and conducted an average of 18.3 lessons per week ( SD=6.7 , range: 2–33).

**Table 1.** Sociodemographic characteristics of the respondents.

	*N* = 399
**Age**	41.91 ± 10
20–29	54 (13.5)
30–39	112 (28.1)
40–49	126 (31.6)
50–59	96 (24.1)
60–69	11 (2.8)
**Gender**	
Male	68 (17)
Female	330 (82.7)
Non-binary	1 (0.3)
**School group**	
Primary school	189 (47.4)
Secondary school	210 (52.6)
**Workload hours (lessons/week)**	18.34 ± 6.69
<15	108 (27.1)
15–20	112 (28.1)
20–25	99 (24.8)
>25	80 (20.1)

Data are given as *M* ± *SD* or *n* (%).

One non-binary participant (without voice assessment) was excluded from further analyses to maintain data privacy. Furthermore, two recordings of the voice assessment were excluded due to extraneous noise that compromised data quality.

### Engagement with the ReSt app

**Figure 1.**
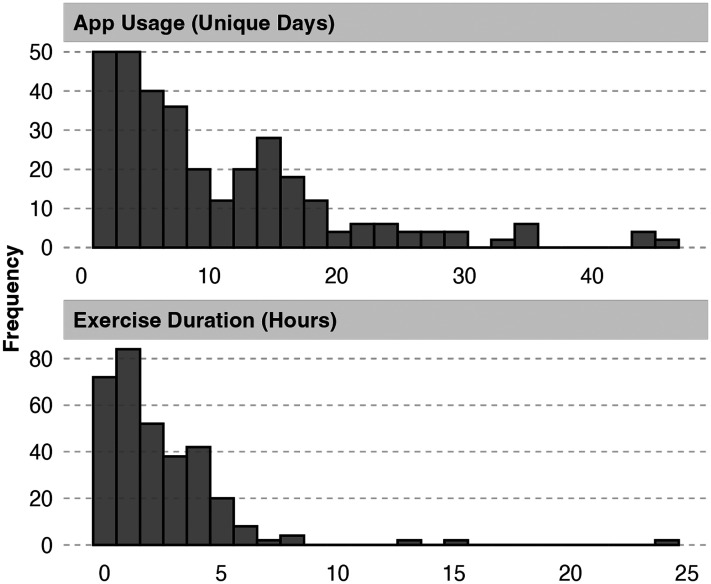
Frequency distributions of app usage (unique days) and exercise duration (hours).

A total of 40 participants (including 10 with voice assessment) opened the ReSt app only once, during initial registration, and were thus excluded from further analyses. Among active users, engagement with the app exhibited considerable heterogeneity (see Figure 1). Temporal engagement showed significant variation, with participants opening exercises in the app between 1 and 45 days. The average number of days spent opening exercises in the app was 10.57 days ( SD=9.07 ), which highlights substantial individual differences in sustained interaction. Whereas some users maintained consistent engagement throughout the study period, others demonstrated intermittent or minimal use. Similarly, time spent on exercise-related app screens varied widely, with participants averaging 2.38 hours ( SD=2.79 ), ranging from as little as 24 seconds to as much as 24.14 hours. Several highly engaged participants not only dedicated significant time to the app but also intensively explored its features, systematically completing all exercises.

### Main analyses

**Figure 2.**
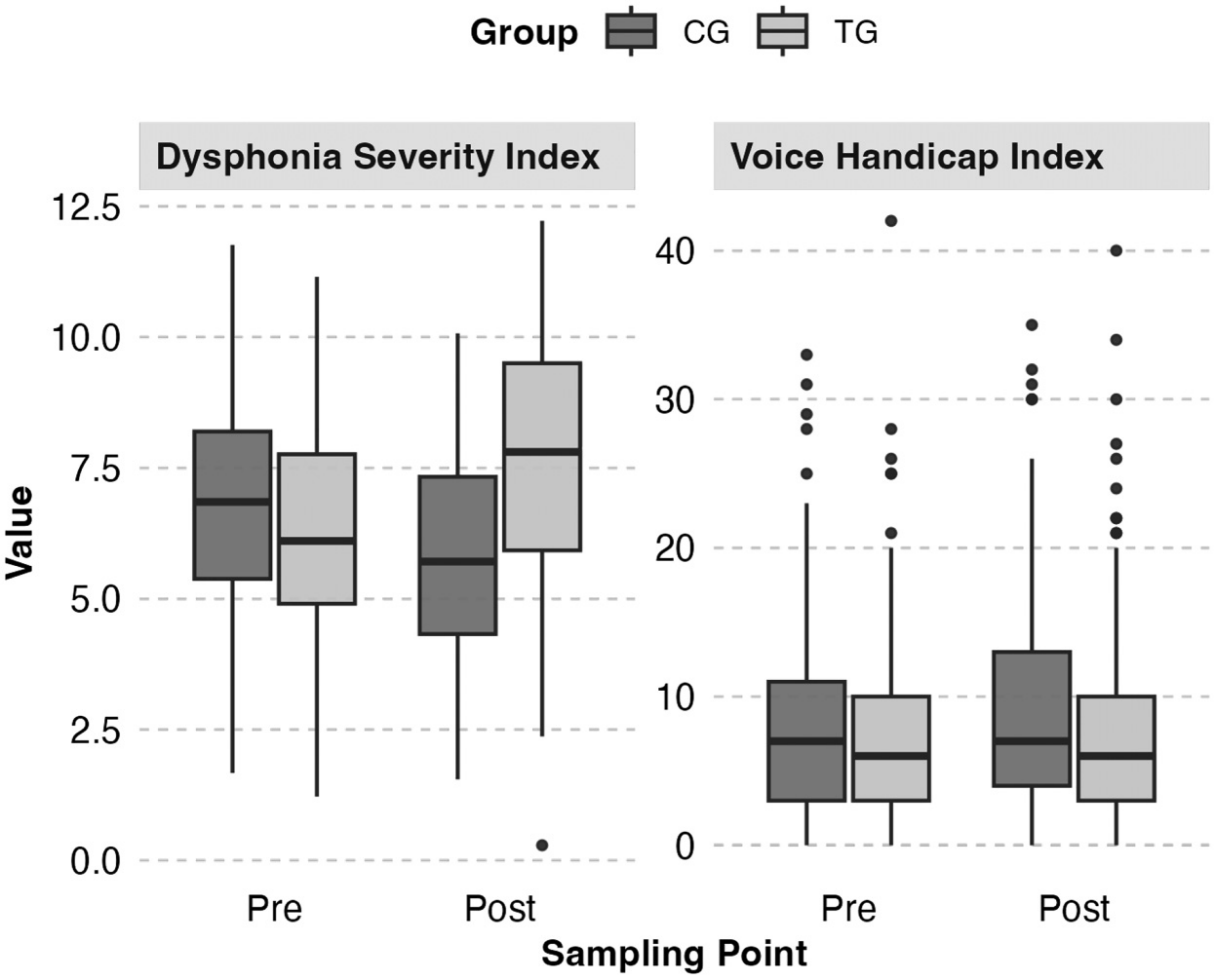
Distribution of Dysphonia Severity Index (DSI) and Voice Handicap Index (VHI) scores at pre-test and post-test. *Note:* CG: control group; TG: training group.

DSI distributions exhibited consistent properties between sampling points, with the pre-test ( M=6.53 , SD=2.15 , Mdn=6.54 ) and post-test ( M=6.61 , SD=2.29 , Mdn=6.39 ) samples showing near-normal distributions. Figure 2 shows the pre-test and post-test DSI scores for both the TG and CG.

The first mixed model analysis examined the effects of the training on DSI scores (see Table 2). A significant interaction was found between sampling points (pre-test to post-test) and groups (TG to CG), yielding a positive coefficient ( γ=2.22,SE=0.32,p<.001 ). This indicates a mean improvement in DSI scores in the TG compared to the CG, supporting the training’s efficacy in enhancing vocal health parameters. Further emphasizing the effectiveness of the intervention, CG had lower DSI scores at the post-test compared to the pre-test ( γ=−0.90,SE=0.21,p<.001 ). Furthermore, older age and a higher number of lessons per week were associated with lower DSI scores (age: γ=−0.03,SE=0.01,p=.034 ; number of lessons: γ=−0.05,SE=0.02,p=.017 ). However, these effects were not found to be statistically significant for α<0.01 . Gender remained a significant predictor, with female participants demonstrating elevated DSI values ( γ=1.34, SE=0.39,p<.001 ).

**Table 2.** Mixed model results for the Dysphonia Severity Index (DSI) with 315 observations from 181 individuals.

Fixed effects	γ	*SE*	99% CI	*t*	*p*
(Intercept)	7.78	0.73	5.89 to 9.66	10.72	<**.001**
sp	−0.90	0.21	−1.45 to −0.36	−4.31	<**.001**
group	−0.47	0.31	−1.27 to 0.34	−1.51	.132
age	−0.03	0.01	−0.07 to 0.01	−2.14	.034
gender	1.34	0.39	0.31 to 2.36	3.40	<**.001**
nr. of lessons	−0.05	0.02	−0.11 to 0.00	−2.41	.017
sp:group	2.22	0.32	1.40 to 3.05	7.06	<**.001**
Random effects	Variance	*SD*			
Participant	2.42	1.56			
Residual	1.74	1.32			
Model fit ( $R^{2}$ )	Marginal	Conditional			
	.17	.65			

*Note:* The higher the DSI value, the higher the voice capability; γ = regression coefficient (not standardized); CI: confidence interval; SD: standard deviation; SE: standard error; the coding of the variables is as follows: **gender**: 0 = male, 1 = female; **sp** (sampling point): 0 = pre-test; 1 = post-test; **group**: 0 = control group; 1 = training group. Statistically significant values are bolded.

**Model equation:** dsi ∼ age + gender + nr. of lessons + sp + group + (1 | participant).

Secondary analysis focusing exclusively on TG participants (see Table 3) revealed one significant correlation between application engagement metrics and DSI outcomes. The more days a teacher opened exercises in the app, the lower the DSI score was from pre-test to post-test voice assessment in the TG.

**Table 3.** Mixed model results for the Dysphonia Severity Index (DSI) with 140 observations from 80 individuals of the training group.

Fixed effects	γ	*SE*	99% CI	*t*	*p*
(Intercept)	7.95	1.18	4.82 to 11.08	6.72	<**.001**
age	−0.03	0.02	−0.10 to 0.03	−1.29	.201
gender	0.35	0.65	−1.36 to 2.07	0.55	.586
nr. of lessons	−0.03	0.03	−0.12 to 0.06	−0.91	.368
sp	1.97	0.34	1.08 to 2.87	5.85	<**.001**
app hours	0.35	0.19	−0.16 to 0.85	1.82	.073
app days	−0.08	0.05	−0.21 to 0.04	−1.75	.083
sp:app hours	0.29	0.15	−0.11 to 0.70	1.92	.060
sp:app days	−0.12	0.04	−0.23 to −0.02	−3.04	**.003**
Random effects	Variance	*SD*			
Participant	3.17	1.78			
Residual	1.30	1.14			
Model fit ( $R^{2}$ )	Marginal	Conditional			
	.21	.77			

*Note:* The higher the DSI value, the higher the voice capability; γ = regression coefficient (not standardized); CI: confidence interval; SD: standard deviation; SE: standard error; the coding of the variables is as follows: **gender**: 0 = male, 1 = female; **sp** (sampling point): 0 = pre-test; 1 = post-test; **group**: 0 = control group; 1 = training group. Statistically significant values are bolded.

**Model equation:** dsi ∼ age + gender + nr. of lessons + sp * app hours + sp * app days + (1 | participant).

Regarding the VHI-12 distributions, the pre-test categorized participants as follows: normal vocal function (51.7%), mild impairment (32.1%), moderate impairment (11.9%), and severe impairment (4.3%). The post-test distribution maintained comparable proportions: 51.8% normal, 28.9% mild, 14.2% moderate, and 5.1% severe impairment.

VHI-12 distributions exhibited positive skewness at both sampling points. Pre-test ( M=7.76 , SD=6.42 , Mdn=6.00 ) and post-test data ( M=8.33 , SD=6.95 , Mdn=6.00 ) were concentrated in lower ranges. Figure 2 shows the pre-test and post-test VHI-12 scores for both the TG and CG.

Despite violation of normality assumptions, comparative mixed linear modeling was conducted for VHI-12 (see Table 4). The interaction between the sampling point and group did not reach statistical significance ( γ=−0.39,SE=0.46,p=.388 ), indicating that there was no clear difference in the trajectories of self-reported voice health between TG and CG.

**Table 4.** Mixed model results for the Voice Handicap Index (VHI-12) with 684 observations from 352 individuals.

Fixed effects	γ	*SE*	99% CI	*t*	*p*
(Intercept)	7.38	1.84	2.62 to 12.14	4.02	<**.001**
sp	0.72	0.31	−0.08 to 1.53	2.33	.020
group	−0.61	0.72	−2.47 to 1.24	−0.86	.392
age	0.02	0.04	−0.07 to 0.11	0.56	.577
gender	−1.05	0.91	−3.41 to 1.31	−1.15	.249
nr. of lessons	0.04	0.05	−0.10 to 0.17	0.76	.448
sp:group	−0.39	0.46	−1.58 to 0.79	−0.86	.388
Random effects	Variance	*SD*			
Participant	35.67	5.97			
Residual	8.66	2.94			
Model fit ( $R^{2}$ )	Marginal	Conditional			
	.01	.81			

*Note:* The higher the VHI value, the more negative consequences of the voice impairment; γ = regression coefficient (not standardized); CI: confidence interval; SD: standard deviation; SE: standard error; the coding of the variables is as follows: **gender**: 0 = male, 1 = female; **sp** (sampling point): 0 = pre-test; 1 = post-test; **group**: 0 = control group; 1 = training group. Statistically significant values are bolded.

**Model equation:** vhi-12 ∼ age + gender + nr. of lessons + sp + group + (1 | participant).

Subsequent analysis of TG participants (see Table 5) revealed a significant interaction between exercise engagement duration and sampling point ( γ=−0.70, SE=0.21,p<.001 ), suggesting negative association between application utilization and VHI-12 outcomes: the more hours a person spent on average exercising in the app, the more their VHI-12 score decreased. In addition, people with higher pre-test VHI-12 scores spent more time on the exercise pages in the app ( γ=0.83, SE=0.31,p=.007 ).

**Table 5.** Mixed model results for the Voice Handicap Index (VHI-12) with 316 observations from 163 individuals of the training group.

Fixed effects	γ	*SE*	99% CI	*t*	*p*
(Intercept)	7.91	2.55	1.28 to 14.55	3.11	**.002**
age	−0.03	0.05	−0.17 to 0.10	−0.63	.527
gender	0.97	1.33	−2.50 to 4.45	0.73	.465
nr. of lessons	0.03	0.08	−0.18 to 0.23	0.35	.726
sp	0.73	0.58	−0.79 to 2.25	1.26	.211
app hours	0.83	0.31	0.03 to 1.63	2.71	**.007**
app days	−0.22	0.09	−0.47 to 0.02	−2.36	.019
sp:app hours	−0.70	0.21	−1.25 to −0.16	−3.38	<**.001**
sp:app days	0.12	0.06	−0.05 to 0.29	1.91	.058
Random effects	Variance	*SD*			
Participant	34.43	5.87			
Residual	10.53	3.24			
Model fit ( $R^{2}$ )	Marginal	Conditional			
	.03	.77			

*Note:* The higher the VHI value, the more negative consequences of the voice impairment; γ = regression coefficient (not standardized); CI: confidence interval; SD: standard deviation; SE: standard error; the coding of the variables is as follows: **gender**: 0 = male, 1 = female; **sp** (sampling point): 0 = pre-test; 1 = post-test; **group**: 0 = control group; 1 = training group. Statistically significant values are bolded.

**Model equation:** vhi-12 ∼ age + gender + nr. of lessons + sp * app hours + sp * app days + (1 | participant).

### Usability analysis

The mean overall MAUQ score was 80.26 ( SD=19.08 ), indicating a generally positive perception of the app’s usability among participants. The subscale for ease of use had a mean score of 25.68 ( SD=5.45 ), suggesting that users found the app relatively easy to use and were generally satisfied with its performance. The interface and satisfaction subscale yielded a mean score of 30.18 ( SD=8.55 ), reflecting moderate satisfaction with the app’s organization of information. Lastly, the usefulness subscale yielded a mean score of 24.40 ( SD=7.60 ), indicating that participants generally perceived the app as beneficial, although there was notable variability in the perceived usefulness. These findings are comparable to other mHealth applications^70–73^ with the exception of item number 9, “I feel comfortable using this app in social settings.” However, this result is not surprising because many of these exercises require loud breathing or sometimes loud phonation. Overall, the app was well received by users and demonstrated strong usability and performance across all three domains measured by the MAUQ (see the supplemental material for more details).

## Discussion

This study examined the effectiveness of the ReSt mobile phone application in managing vocal health among German school teachers. The MAUQ scores indicate that the app was well received and was effectively designed to support users in their vocal health management. Importantly, the positive usability scores also indicate that the results are unlikely to be distorted by frustration or dissatisfaction with the app. This strengthens the reliability of the findings, as reduced usage of the app due to a lack of usability might otherwise have influenced the study’s conclusions.

For the evaluation we employ both objective (DSI) and subjective (VHI-12) measures. At the outset, it is crucial to contextualize the baseline vocal performance of our participants. The mean pre-test DSI score of 6.53 notably exceeds the previously reported population norm of 3.05, as established in Sobol and Sielska-Badurek’s meta-analysis of 1330 healthy subjects.^74^ This elevated baseline may be attributed to the professional vocal demands placed on teachers, who frequently use their voices in classroom settings, thereby enhancing their voice capability. Much as physical exercise strengthens muscles, regular vocal projection in educational settings appears to enhance voice capability.

Our study revealed a notable discrepancy between quantitative and qualitative assessments of voice health. The DSI and VHI-12 yielded substantially divergent outcomes, contradicting previous hypotheses regarding their similarity.^56,75^

The DSI results showed a significant growth difference of 2.22 between the CG and the TG. The TG exhibited enhanced vocal parameters whereas the CG demonstrated deterioration between initial and subsequent assessment. Considering that the DSI has been designed so that − 5 represents a severe case of dysphonia and + 5 represents a normal voice,^56^ this improvement represents a meaningful variation. The magnitude approximates the previously established perceptible threshold of 2.49 points,^76^ suggesting a tangible impact of the training. Our analysis revealed significantly reduced DSI values among male teachers, aligning with several studies^17,77,78^ but contradicting investigations reporting gender independence^56,79–82^ or inverse associations for younger participants.^83^

Conversely, VHI-12 analysis revealed no statistically significant inter-group variations due to the ReSt app, with gender remaining a non-discriminating factor. This incongruence emphasizes the multifaceted nature of voice assessments. These findings challenge the presumed equivalence between objective and subjective vocal health metrics. Whereas previous investigations utilized the comprehensive 30-item VHI for DSI criterion validation,^56^ our results indicate the necessity for more comprehensive evaluation protocols.

The VHI-12 predominantly assesses psychosocial implications of vocal dysfunction, whereas the DSI provides quantitative analysis of voice capability. This methodological distinction is supported by existing literature.^84–86^

Furthermore, the two instruments operate on distinct temporal frameworks. The DSI provides instantaneous voice assessment, whereas the VHI-12 evaluates perceived impairment over an extended, undefined period. This temporal disparity presents methodological challenges in intervention studies with condensed measurement intervals, as participants must retrospectively evaluate experiences spanning weeks or months. Moreover, the VHI-12 measures secondary manifestations rather than primary vocal pathology, introducing a temporal lag between dysfunction onset and its implications. In this context, while the ReSt app was designed for prolonged use to maximize efficacy, the study encompasses a relatively abbreviated time window. This limited duration may not allow sufficient time for vocal improvements to manifest in ways that would be captured by the VHI-12, suggesting that extended app usage beyond the study period might yield measurable changes as more time passes to allow for manifestation of improvements.

The implementation of VHI-12 in preventative research involving predominantly normophonic participants is constrained by pronounced floor effects. The predominance of minimal scores limits the potential for demonstrable improvement between assessments. Consequently, whereas the VHI-12 demonstrates utility as a clinical screening instrument for voice disorders, it proves suboptimal in the context of prevention studies. The DSI’s continuous measurement scale appears more appropriate for this investigative context. A first, albeit partial, complement to the VHI-12 in intervention studies is provided by the German questionnaire on voice self-concept (*Fragebogen zur Erfassung des Stimmlichen Selbstkonzepts*).^87^

Analysis of log data revealed just one counterintuitive significant relationship between duration of exercise engagement and change in DSI within the TG, suggesting that the effectiveness of the training was not necessarily dependent on the amount of engagement. Analysis of the VHI-12 data reveals a significant association between usage patterns and perceived voice-related impairment. The results show an inverse relationship between time spent interacting with the application’s exercises and VHI-12 scores, with lower scores indicating less voice-related psychosocial impact. In addition, teachers with higher initial VHI-12 scores spent more time interacting with the application’s exercises. This finding suggests that participants experiencing more severe consequences of voice problems invested greater effort in the training.

Multiple factors potentially account for these disparate correlations, particularly regarding the DSI measurements. The recorded interface engagement metrics may inadequately capture genuine participation, as the system cannot differentiate between periods of user inactivity, cursory engagement, and meaningful execution of prescribed exercises. This is well-documented in mHealth research.^88–91^

Furthermore, the inherent simplicity and memorability of the application’s exercises may have allowed participants to internalize them after minimal time, facilitating independent practice without digital assistance. Such autonomous implementation, although aligned with the application’s goal of integrating vocal health practices into everyday routines, remains unquantifiable through standard usage metrics.^89,90^

The observed results may reflect heterogeneity among participants. This may relate to differences in vocal self-concept and knowledge of their vocal deficits, which could potentially modulate the time investment required for beneficial training. More skilled teachers may require shorter training periods, although this relationship remains to be investigated. Additionally, in the context of mHealth interventions, the influence of technology familiarity has often been discussed in the literature as a moderating factor.^92–95^ Taken together, these hypothesized factors could result in the effectiveness of time invested in training being highly dependent on individual prerequisites, which may explain why no clear effect was found regarding app engagement. Further research is needed to systematically examine these factors.

## Limitations

Our study focused on a specific population consisting of German school teachers, which introduces several potential limitations. The geographic and occupational specificity of the sample limits the generalizability of the findings. These results may be confined to the German educational context and may not be directly applicable to other education systems. Additionally, whereas the focus on teachers provides valuable insights into the experiences of this group as occupational voice users, it precludes drawing conclusions about other professions with distinct vocal demands. In addition, self-selection bias among participants who choose to participate in the study and especially in the in-person voice assessment, potential differences between participants and non-participants, and lack of demographic diversity within the sample may introduce potential biases.

The mobile application intervention presents several methodological considerations. Although app usage was tracked, comprehensive contextual data regarding participants’ daily vocal training outside of the app could not be recorded.

## Conclusion

Our results demonstrated that the mobile app intervention had a significant positive impact on teachers’ vocal capability, marking an important advancement in voice disorder prevention approaches. Although a previous systematic review by Ramos et al.^50^ found inconclusive evidence for traditional direct and indirect voice training methods, our mobile app-based intervention presents a novel and promising direction. Interestingly, the ReSt app showed no significant effect on subjective voice health measures, but objective voice parameters improved remarkably after 46 days of training, highlighting that subjective and objective measures are not directly interchangeable but rather complementary aspects of voice health assessment. The success of our digital intervention suggests that leveraging modern technology can bridge the gap between theoretical voice health knowledge and practical implementation in teachers’ daily routines.

## Supplemental Material

sj-pdf-1-dhj-10.1177_20552076251351805 - Supplemental material for Effectiveness of a mobile phone application in managing voice health for teachers: A randomized controlled trial Supplemental material, sj-pdf-1-dhj-10.1177_20552076251351805 for Effectiveness of a mobile phone application in managing voice health for teachers: A randomized controlled trial by Jonas Hauck, Christian Gegner, Marina Giglberger and Sven Hilbert in DIGITAL HEALTH

## Notes

1. https://www.uni-regensburg.de/ethikkommission/startseite/index.html.
