# Supplementary material for: Effectiveness of a mobile phone application in managing voice health for teachers: A randomized controlled trial
Source: Digit Health. 2025 Jul 2;11:20552076251351805. doi: 10.1177/20552076251351805 (PMC12227945; doi:10.1177/20552076251351805)
Supplement: sj-pdf-1-dhj-10.1177_20552076251351805 - Supplemental material for Effectiveness of a mobile phone application in managing voice health for teachers: A randomized controlled trial [file sj-pdf-1-dhj-10.1177_20552076251351805.pdf]

Supplemental material

The datasets used and the code used for analysis are available from the OSF repository.

MAUQ Items	Mean (SD)
**Ease of use**	**25.4 (5.60)**
MAUQ_SQ01. The app was easy to use.	5.18 (1.27)
MAUQ_SQ02. It was easy for me to learn to use the app.	5.40 (1.21)
MAUQ_SQ03. The navigation was consistent when moving between screens.	5.28 (1.21)
MAUQ_SQ04. The interface of the app allowed me to use all the functions (such as entering information, responding to reminders, viewing information) offered by the app.	4.90 (1.35)
MAUQ_SQ05. Whenever I made a mistake using the app, I could recover easily and quickly.	4.87 (1.53)
**Interface and satisfaction**	**29.88 (8.58)**
MAUQ_SQ06. I like the interface of the app.	4.81 (1.45)
MAUQ_SQ07. The information in the app was well organized, so I could easily find the information I needed.	4.70 (1.53)
MAUQ_SQ08. The app adequately acknowledged and provided information to let me know the progress of my action.	4.50 (1.75)
MAUQ_SQ09. I feel comfortable using this app in social settings.	2.72 (2.07)
MAUQ_SQ10. The amount of time involved in using this app has been fitting for me.	3.64 (1.81)
MAUQ_SQ11. I would use this app again.	4.89 (1.65)
MAUQ_SQ12. Overall, I am satisfied with this app.	4.91 (1.48)
**Usefulness**	**24.3 (7.60)**
MAUQ_SQ13. The app would be useful for my health and well-being.	4.83 (1.53)
MAUQ_SQ14. The app improved my access to health care services.	2.98 (2.05)
MAUQ_SQ15. The app helped me manage my health effectively.	4.01 (1.71)
MAUQ_SQ16. This app has all the functions and capabilities I expected it to have.	4.41 (1.58)
MAUQ_SQ17. I could use the app even when the Internet connection was poor or not available.	4.26 (1.63)
MAUQ_SQ18. This mHealth app provided an acceptable way to receive health care services, such as accessing educational materials, tracking my own activities, and performing self-assessment.	3.92 (1.71)

Item Code	German translation
MAUQ_SQ01	Die App war einfach zu bedienen
MAUQ_SQ02	Für mich war die Bedienung der App leicht zu erlenen
MAUQ_SQ03	Die Navigation war einheitlich
MAUQ_SQ04	Die Benutzeroberfläche der App hat es mir ermöglicht, alle von der App angegebenen Funktionen zu nutzen (z.B. Eingabe von Informationen, Beantwortung von Erinnerungen, Anzeige von Informationen)
MAUQ_SQ05	Wenn ich bei der Verwendung der App einen Fehler gemacht habe, konnte ich ihn leicht und schnell beheben
MAUQ_SQ06	Mir gefällt die Benutzeroberfläche der App
MAUQ_SQ07	Die Inhalte der App waren gut organisiert, so dass ich die benötigten Informationen leicht finden konnte
MAUQ_SQ08	Die App hat mich angemessen über meinen Fortschritt informiert
MAUQ_SQ09	Ich fühle mich wohl, wenn ich die App in der Öffentlichkeit benutze
MAUQ_SQ10	Für mich war der Zeitaufwand für die Nutzung dieser App angemessen
MAUQ_SQ11	Ich würde diese App wieder benutzen
MAUQ_SQ12	Insgesamt bin ich mit dieser App zufrieden
MAUQ_SQ13	Die App ist nützlich für meine Gesundheit und mein Wohlbefinden
MAUQ_SQ14	Die App hat meinen Zugang zu Gesundheitsdienstleitungen verbessert
MAUQ_SQ15	Die App mich effektiv bei der Gesunderhaltung unterstützt
MAUQ_SQ16	Die App hat alle Funktionen und Möglichkeiten, die ich erwartet habe
MAUQ_SQ17	Ich konnte die App auch dann nutzen, wenn die Internetverbindung schlecht verfügbar war
MAUQ_SQ18	Diese App bot mir eine gute Möglichkeit, Gesundheitsdienste in Anspruch zu nehmen, wie z.B. den Zugang zu Bildungsmaterialien, die Verfolgung meiner eigenen Handlungen und die Durchführung von Selbsteinschätzungen
